# Topological data analysis to identify cardiac resynchronization therapy patients exhibiting benefit from an implantable cardioverter-defibrillator

**DOI:** 10.1007/s00392-023-02281-6

**Published:** 2023-08-25

**Authors:** Boglárka Veres, Walter Richard Schwertner, Márton Tokodi, Ádám Szijártó, Attila Kovács, Eperke Dóra Merkel, Anett Behon, Luca Kuthi, Richárd Masszi, László Gellér, Endre Zima, Levente Molnár, István Osztheimer, Dávid Becker, Annamária Kosztin, Béla Merkely

**Affiliations:** Heart and Vascular Center, Semmelweis University, Városmajor Str. 68, 1122 Budapest, Hungary

**Keywords:** Heart failure, Cardiac resynchronization therapy, Implantable cardioverter-defibrillator, Personalized medicine, Topological data analysis

## Abstract

**Background:**

Current guidelines recommend considering multiple factors while deciding between cardiac resynchronization therapy with a defibrillator (CRT-D) or a pacemaker (CRT-P). Nevertheless, it is still challenging to pinpoint those candidates who will benefit from choosing a CRT-D device in terms of survival.

**Objective:**

We aimed to use topological data analysis (TDA) to identify phenogroups of CRT patients in whom CRT-D is associated with better survival than CRT-P.

**Methods:**

We included 2603 patients who underwent CRT-D (54%) or CRT-P (46%) implantation at Semmelweis University between 2000 and 2018. The primary endpoint was all-cause mortality. We applied TDA to create a patient similarity network using 25 clinical features. Then, we identified multiple phenogroups in the generated network and compared the groups’ clinical characteristics and survival.

**Results:**

Five- and 10-year mortality were 43 (40–46)% and 71 (67–74)% in patients with CRT-D and 48 (45–50)% and 71 (68–74)% in those with CRT-P, respectively. TDA created a circular network in which we could delineate five phenogroups showing distinct patterns of clinical characteristics and outcomes. Three phenogroups (1, 2, and 3) included almost exclusively patients with non-ischemic etiology, whereas the other two phenogroups (4 and 5) predominantly comprised ischemic patients. Interestingly, only in phenogroups 2 and 5 were CRT-D associated with better survival than CRT-P (adjusted hazard ratio 0.61 [0.47–0.80], p < 0.001 and adjusted hazard ratio 0.84 [0.71–0.99], p = 0.033, respectively).

**Conclusions:**

By simultaneously evaluating various clinical features, TDA may identify patients with either ischemic or non-ischemic etiology who will most likely benefit from the implantation of a CRT-D instead of a CRT-P.

**Graphical abstract:**

Topological data analysis to identify phenogroups of CRT patients in whom CRT-D is associated with better survival than CRT-P. *AF* atrial fibrillation, *CRT* cardiac resynchronization therapy, *CRT-D* cardiac resynchronization therapy defibrillator, *CRT-P* cardiac resynchronization therapy pacemaker, *DM* diabetes mellitus, *HTN* hypertension, *LBBB* left bundle branch block, *LVEF* left ventricular ejection fraction, *MDS* multidimensional scaling, *MRA* mineralocorticoid receptor antagonist, *NYHA* New York Heart Association

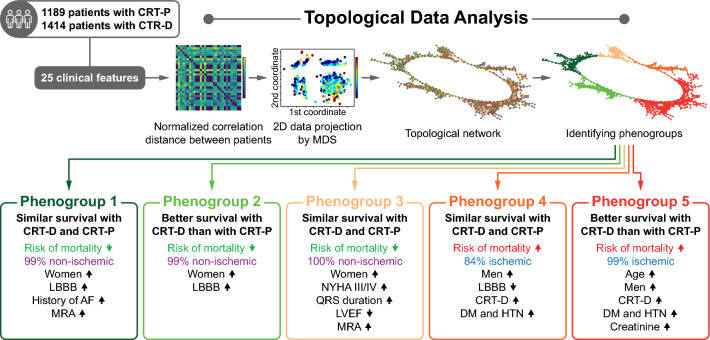

**Supplementary Information:**

The online version contains supplementary material available at 10.1007/s00392-023-02281-6.

## Introduction

Cardiac resynchronization therapy (CRT) is a standard device therapy in a selected subgroup of patients with symptomatic heart failure, reduced left ventricular ejection fraction (LVEF), and prolonged QRS duration in sinus rhythm [1–3]. Despite the well-defined indications for CRT implantation, it is still challenging to pinpoint those candidates who would benefit from an additional implantable cardioverter-defibrillator (ICD) [4]. There is growing scientific evidence that ischemic etiology is one of the most important factors to be considered while deciding between the implantation of a CRT-defibrillator (CRT-D) or a CRT-pacemaker (CRT-P) [5, 6]. Nevertheless, the current guidelines of the European Society of Cardiology (ESC) recommend the simultaneous evaluation of etiology and multiple risk factors of sudden cardiac death (SCD) and all-cause mortality, such as age, presence of myocardial fibrosis, and comorbidities [4]. It is also emphasized that this risk assessment should be carried out in a personalized manner, in which novel and robust data analysis techniques could have a pivotal role as they could be tailored for the integrated assessment of risk profiles.

One such technique is topological data analysis (TDA) which has gained increasing popularity within the realm of cardiovascular medicine [7–9]. By synthesizing multiple domains of input features using the tools of topology, TDA enables the encoding of complex relationships within medical datasets in a simple and compressed format (i.e., as patient similarity networks) [10]. Then, through the visual interpretation and statistical analysis of the generated networks, we can identify specific subgroups (i.e., phenogroups) of patients within monolithic disease categories that might respond differently to a given therapy. Thus, we may assume that TDA could be used to improve risk stratification and optimize device selection in CRT candidates.

Accordingly, we used TDA to identify phenogroups of CRT patients in whom CRT-D is associated with better survival than CRT-P.

## Methods

### Study population and data collection

A total of 2603 patients with heart failure and reduced LVEF (New York Heart Associaton [NYHA] II–IVa) and a prolonged QRS duration (QRS ≥ 130 ms) who underwent successful CRT implantation at the Heart and Vascular Center of Semmelweis University (Budapest, Hungary) between October 2000 and September 2018 were included in our retrospective database. CRT devices were implanted as per guidelines [4]. Device implantation was performed using standard transvenous techniques under local anesthesia. Baseline clinical characteristics, such as demographics, medical history, cardiovascular risk factors, physical status, medications, echocardiographic, and laboratory results, were retrieved for each patient from the electronic medical record system of Semmelweis University. The study protocol complies with the Declaration of Helsinki and was approved by the Semmelweis University Regional and Institutional Committee of Science and Research Ethics (approval number: 161/2019). Obtaining informed consent was waived owing to the retrospective nature of the analysis.

### Outcomes of interest

The primary endpoint was all-cause mortality. Status (i.e., dead or alive) and the date of death were obtained for all patients by querying the National Health Insurance Database of Hungary in May 2021. We also performed a sensitivity analysis using the composite of all-cause death, heart transplantation, and left ventricular assist device implantation.

### Topological data analysis

TDA is an emerging technique that adopts methods from the field topology, a discipline of mathematics focusing on shape analysis, to create compact visual representations of high-dimensional datasets in an unsupervised fashion. It dwells on the concept that shape analysis techniques can be applied to connect data points (such as patients) with similar characteristics in a multidimensional space and to plot these connections as a two-dimensional topological network. The generated network consists of nodes and edges. Nodes represent collections of patients with similar characteristics, whereas edges connect two nodes if they have at least one patient in common. Networks can be color-coded based on outcomes to gain insights into the data. Before generating a topological network, the following parameters are needed to be defined: (1) a distance metric, which measures the similarity between data points, and (2) one or more lenses, which are filter functions describing the distribution of the data. For each lens, we also have to set the gain (which controls the number of nodes) and resolution (which controls the number of edges) prior to analysis.

After discarding features with 40% or more missing values (Supplemental Table 1), we used the remaining twenty-five to generate the topological network: age, sex, type of the implanted device, etiology of heart failure, NYHA functional class, history of atrial fibrillation, history of ventricular arrhythmia, history of diabetes mellitus, history of hypertension, body mass index, history of myocardial infarction (MI), history of percutaneous coronary intervention (PCI), history of coronary artery bypass graft surgery (CABG), serum creatinine, serum urea, serum sodium, hemoglobin, LVEF, left ventricular end-diastolic and end-systolic diameters, QRS morphology (as the presence of left bundle branch block [LBBB]), usage of beta-blockers, mineralocorticoid receptor antagonists (MRAs), amiodarone, and oral anticoagulants. Missing values of the features were replaced using mean imputation, which was followed by Z-score transformation. We applied normalized correlation as the distance metric in conjunction with two multidimensional scaling lenses (with a resolution of 29 and a gain of 1.6, both equalized). Patients placed into nodes disconnected from the main network (n = 244) were considered outliers and were omitted from the further steps of the analysis.

After creating the topological network, we wanted to divide it into regions with distinct clinical characteristics and all-cause mortality rates. To this end, we first performed community autogrouping using the Louvain method, which finds the best possible grouping of nodes with high intra-group but low inter-group connectivity fields [11]. This step resulted in the generation of 16 autogroups. Next, the group with the most outbound connections relative to its size (i.e., the number of nodes in the group) was merged with an adjacent group to which it was connected with the most edges. This step was repeated multiple times until five densely connected groups of nodes (referred to as phenogroups) were created. Due to the inherent nature of TDA, the phenogroups overlapped partially (i.e., 16 patients belonged to two phenogroups at the same time). However, this phenomenon does not violate any assumptions or requirements of the statistical tests used for subgroup comparisons.

TDA and autogrouping were performed using the EurekaAI Workbench (version 3.1.0, SymphonyAI, Palo Alto, California, USA) and the EurekaAI Python software development kit (version 3.1.0, SymphonyAI, Palo Alto, California, USA).

### Statistical analysis

Statistical analysis was performed using GraphPad Prism (version 8.0, GraphPad Software, San Diego, California, USA), SPSS (version 21.0, IBM, Armonk, New York, USA), and R (version 4.1.2, R Foundation for Statistical Computing, Vienna, Austria). Continuous variables with a normal distribution are presented as mean ± standard deviation, whereas those with non-normal distribution are reported as median and interquartile range (IQR). Categorical variables are expressed as frequencies (n) and percentages (%). The clinical characteristics of the CRT-D and CRT-P groups were compared using unpaired Student’s t-test or Mann–Whitney U test for continuous variables and Chi-squared or Fisher’s exact test for categorical variables, as appropriate. The event-free survival of patient subgroups was visualized on Kaplan–Meier curves, and log-rank tests were performed for comparison. Follow-up duration was estimated using the reverse Kaplan–Meier method, and mortality and absolute risk reduction were calculated based on Kaplan–Meier estimates. Absolute risk reduction was considered significant if the value 0 fell outside of its confidence interval. Univariable and multivariable Cox proportional hazards models were used to compute hazard ratios (HRs) with 95% confidence intervals (CIs). Only features with no missing values were considered in the multivariable models. Right censoring was applied if a patient (1) had a subsequent CRT-D upgrade after being implanted with a CRT-P device (n = 65), (2) underwent left ventricular assist device implantation (n = 6), or heart transplantation (n = 41). In the sensitivity analysis, right censoring was only applied in the case of upgrade procedures. The characteristics of the five TDA-derived phenogroups were compared in a pairwise manner using the Kolmogorov–Smirnov test (for continuous variables) and Chi-squared or Fisher’s exact test (for categorical variables), as appropriate. All statistical tests were two-sided, and a p value of < 0.05 was considered statistically significant.

## Results

### Baseline clinical characteristics of the study cohort

Between 2000 and 2018, 1189 patients were implanted successfully with CRT-P (46%) and 1414 with CRT-D (54%) at our center (Table 1). The CRT-D group comprised significantly younger (67 (60–73) vs. 69 (62–76) years, p < 0.001) and less symptomatic patients (NYHA functional class III/IV: 51 vs. 61%, p < 0.001) and a higher proportion of men (81 vs. 67%, p < 0.001). More patients had ischemic etiology (58 vs. 38%, p < 0.001) and experienced ventricular arrhythmias previously (37 vs. 11%, p < 0.001) in the CRT-D group than in the CRT-P group. CRT-D patients had wider QRS complexes (160 (140–170) vs. 160 (150–180) ms, p = 0.003), lower LVEF (28 (23–32) vs. 30 (25–35)%, p < 0.001), and larger LV diameters than CRT-P patients. Beta-blockers (90 vs. 87%, p = 0.018), MRAs (73 vs. 62%, p < 0.001), and amiodarone (32 vs. 21%, p < 0.001) were administered more frequently in the CRT-D group.

**Table 1:** Baseline clinical characteristics of the entire study cohort

	All n = 2603	CRT-P n = 1189	CRT-D n = 1414	p value
Demographics, physical status, key electrophysiological characteristics				
Age, years	68 (61–74)	69 (62–76)	67 (60–73)	< 0.001
Male sex	1947 (75)	798 (67)	1149 (81)	< 0.001
BMI, kg/m^2^ (1581)	28 (25–31)	27 (24–31)	28 (25–31)	0.103
NYHA III-IV (2175)	1213 (56)	611 (61)	602 (51)	< 0.001
LBBB	1822 (70)	841 (71)	981 (69)	0.466
QRS duration, ms (910)	160 (140–180)	160 (150–180)	160 (140–170)	0.003
Medical history				
Atrial fibrillation	984 (38)	471 (40)	513 (36)	0.080
Ventricular arrhythmia	654 (25)	130 (11)	524 (37)	< 0.001
Diabetes mellitus	950 (37)	431 (36)	519 (37)	0.840
Hypertension	1877 (72)	858 (72)	1019 (72)	0.970
Ischemic etiology	1273 (49)	456 (38)	817 (58)	< 0.001
Myocardial infarction	998 (38)	354 (30)	644 (46)	< 0.001
PCI	771 (30)	265 (22)	506 (36)	< 0.001
CABG	349 (13)	99 (8)	250 (18)	< 0.001
Laboratory measurements				
NT-proBNP, pg/mL (348)	2744 (1483–3969)	2829 (1590–3682)	2650 (1434–4167)	0.586
Creatinine, μmol/L (1681)	101 (82–130)	101 (80–132)	102 (83–129)	0.475
eGFR, mL/min/1.73m^2^ (1682)	64 (48–81)	62 (46–81)	65 (49–81)	0.082
BUN, mmol/L (1656)	8 (6–12)	9 (7–12)	8 (6–12)	0.147
Serum sodium, mmol/L (1561)	138 (136–141)	139 (136–141)	138 (136–140)	0.155
Hemoglobin, g/dL (1646)	14 ± 2	14 ± 2	14 ± 2	0.549
Echocardiographic measurements				
LVEDD, mm (1679)	63 (57–70)	62 (56–69)	64 (58–70)	0.003
LVESD, mm (1500)	53 (47–60)	52 (45–59)	54 (48–61)	< 0.001
LVEF, % (1903)	28 (24–33)	30 (25–35)	28 (23–32)	< 0.001
Medications				
ACEI/ARB (2380)	2186 (92)	953 (91)	1233 (93)	0.070
Beta-blockers (2377)	2115 (89)	915 (87)	1200 (90)	0.018
MRA (2377)	1614 (68)	653 (62)	961 (73)	< 0.001
Loop diuretics (2379)	1888 (79)	832 (79)	1056 (80)	0.919
Amiodarone (2368)	641 (27)	215 (21)	426 (32)	< 0.001
OAC (2355)	703 (30)	328 (31)	375 (29)	0.135

The value (in parenthesis) after a feature’s name indicates the number of patients with available data. If there is no value reported, data were available for all patients. Continuous variables are expressed as mean ± standard deviation or median (interquartile range), whereas categorical variables are reported as frequencies (n) and percentages (%). The characteristics of the CRT-P and CRT-D groups were compared using unpaired Student's t-test or Mann–Whitney U test for continuous variables and Chi-squared or Fisher's exact test for categorical variables, as appropriate

*ACEI* angiotensin-converting enzyme inhibitor, *ARB* angiotensin receptor blocker, *BMI* body mass index, *BUN* blood urea nitrogen, *CABG* coronary artery bypass graft surgery, *CRT-D* cardiac resynchronization therapy defibrillator, *CRT-P* cardiac resynchronization therapy pacemaker, *eGFR* estimated glomerular filtration rate, *LBBB* left bundle branch block, *LVEDD* left ventricular end-diastolic diameter, *LVEF* left ventricular ejection fraction, *LVESD* left ventricular end-systolic diameter, *MRA* mineralocorticoid receptor antagonist, *NT-proBNP* N-terminal pro-brain natriuretic peptide, *NYHA* New York Heart Association, *OAC* oral anticoagulant, *PCI* percutaneous coronary intervention

A total of 1273 (49%) patients with ischemic etiology and 1330 (51%) patients with non-ischemic etiology were included in our retrospective database. From the former, 456 (36%) patients were implanted with CRT-P and 817 (64%) with CRT-D, whereas from the latter, 733 (55%) patients were implanted with CRT-P and 597 (45%) with CRT-D. The baseline clinical characteristics of the ischemic and non-ischemic subgroups are presented in Supplemental Tables 2 and 3, respectively.

### All-cause mortality of CRT-D and CRT-P patients

**Fig. 1:**
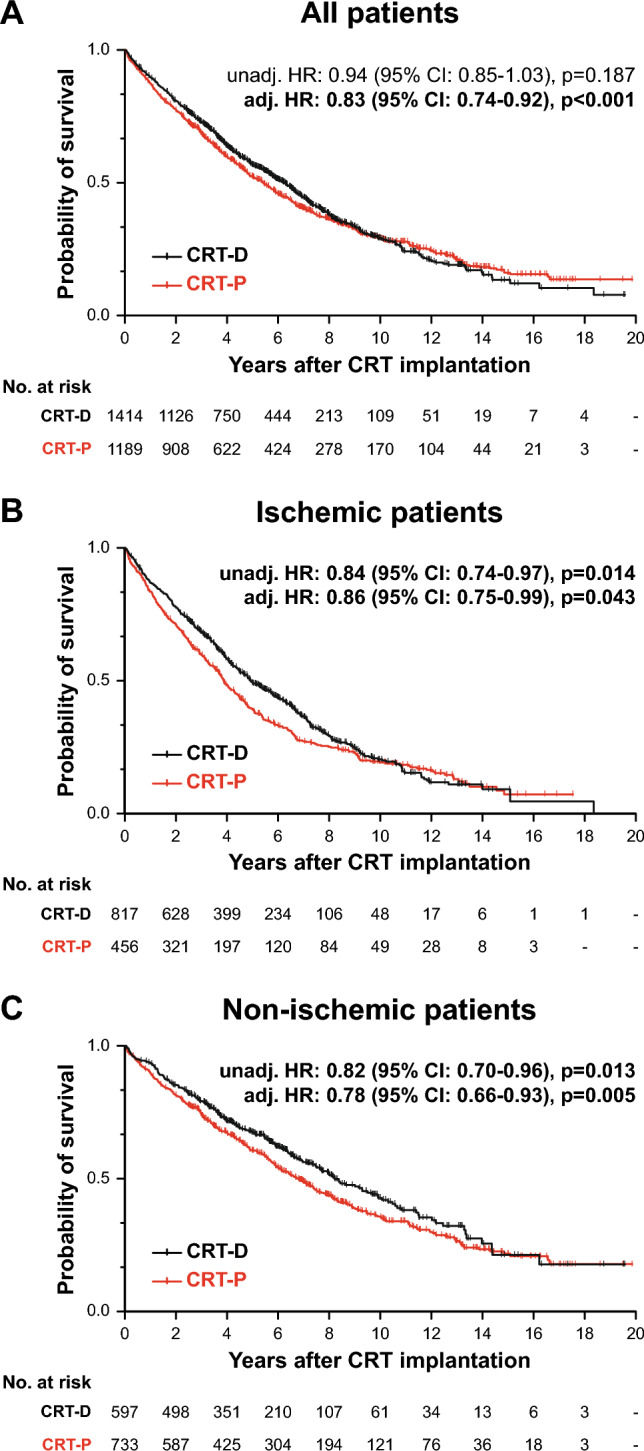
Kaplan–Meier estimates of the time to death from any cause in the entire study cohort, ischemic patients, and non-ischemic patients. Univariable and multivariable Cox proportional hazards models were used to compute hazard ratios with 95% confidence intervals. Each multivariable model included the following features: device type, age, sex, history of atrial fibrillation, and history of ventricular arrhythmia. *CI* confidence interval, *CRT-D* cardiac resynchronization therapy defibrillator, *CRT-P* cardiac resynchronization therapy pacemaker, *HR* hazard ratio

**Fig. 2:**
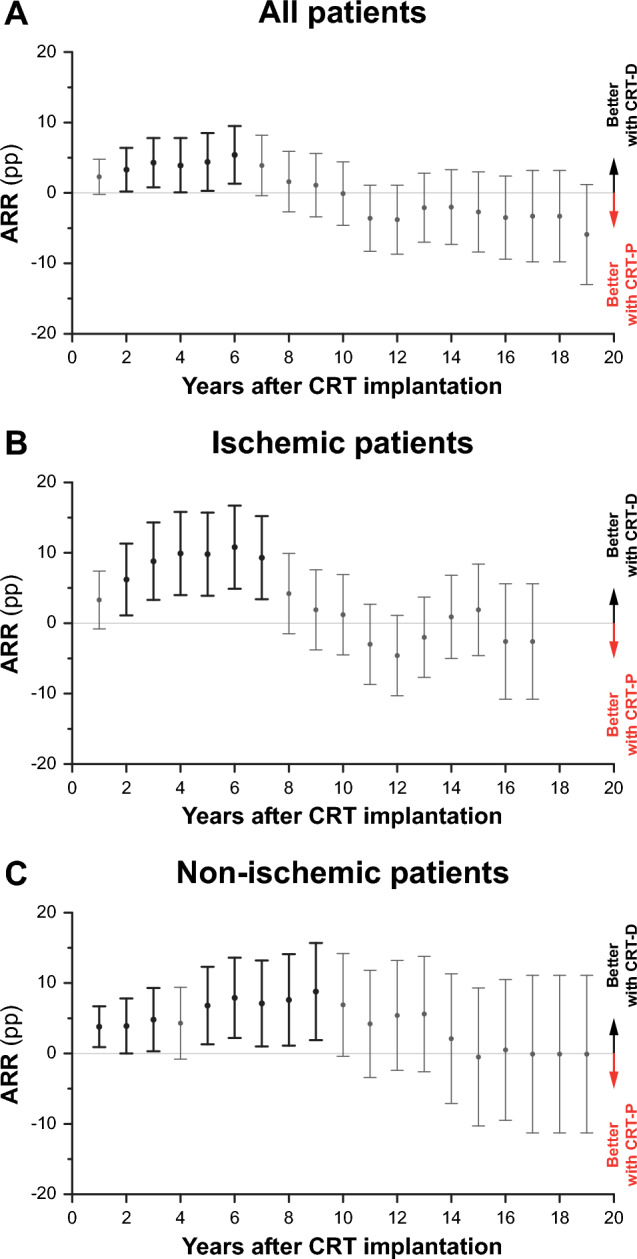
Absolute risk reduction associated with CRT-D vs. CRT-P at each year of the follow-up. Absolute risk reductions and their confidence intervals were calculated based on Kaplan–Meier estimates. Absolute risk reduction was considered significant if the value 0 fell outside of its confidence interval. *ARR* absolute risk reduction, *CRT* cardiac resynchronization therapy, *CRT-D* cardiac resynchronization therapy defibrillator, *CRT-P* pacemaker, *pp* percentage point

Over the median follow-up of 8.0 (5.3–12.1) years, a total of 1572 patients died in our cohort. Based on Kaplan–Meier estimates, 5- and 10-year mortality were 45 (43–47)% and 71 (68–73)% in the entire cohort, 43 (40–46)% and 71 (67–74)% in patients with CRT-D, and 48 (45–50)% and 71 (68–74)% in those with CRT-P, respectively (Supplemental Table 4). CRT-D was not associated with a greater survival benefit than CRT-P in univariable Cox regression (unadjusted HR 0.94, 95% CI 0.85–1.03, p = 0.220). However, after adjustment for relevant clinical covariates (i.e., age, sex, etiology of heart failure, and history of ventricular arrhythmia), CRT-D was superior to CRT-P (adjusted HR 0.83, 95% CI 0.74–0.92, p < 0.001) in terms of all-cause mortality (Fig. 1A). When sequential multivariable models were built by adding these covariates to the univariable model in a stepwise manner (Supplemental Table 5), we found both etiology and history of ventricular arrhythmia to be negative confounding variables (i.e., not adjusting for them would result in the underestimation of the true strength of the association between the type of the implanted device and all-cause mortality). As shown by absolute risk reductions calculated at each year of the follow-up, CRT-D was associated with better survival than CRT-P only in the 2-to-6-year interval after implantation (Fig. 2A, Supplemental Table 4). In the sensitivity analysis, device type was found to be a significant predictor of the composite endpoint in the multivariable (adjusted HR 0.85, 95% CI 0.76–0.95, p = 0.003) but not the univariable Cox model (unadjusted HR 0.97, 95% CI 0.88–1.07, p = 0.498) (Supplemental Fig. 1A).

Among those with ischemic etiology, 882 patients reached the primary endpoint over the median follow-up duration of 7.9 (5.1–12.2) years, and 5- and 10-year mortality were 54 (51–57)% and 80 (77–82)%, respectively (Supplemental Table 4). In this subset of patients, CRT-D was associated with a lower risk of death compared to CRT-P according to both univariable (unadjusted HR 0.84, 95% CI 0.74–0.97, p = 0.014) and multivariable analysis (adjusted HR 0.86, 95% CI 0.75–0.99, p = 0.043) (Fig. 1B). Based on absolute risk reduction, CRT-D was associated with better survival than CRT-P only in the 2-to-7-year interval following implantation (Fig. 2B, Supplemental Table 4). Sensitivity analysis showed that the type of device is a significant predictor of the composite endpoint only in the univariable (unadjusted HR 0.87, 95% CI 0.76–0.99, p = 0.046) but not the multivariable Cox model (adjusted HR 0.88, 95% CI 0.76–1.01, p = 0.071) (Supplemental Fig. 1B).

In patients with non-ischemic etiology, 690 individuals died over 8.4 (5.5–12.0) years. Five-year mortality was 36 (34–39)%, whereas 10-year mortality was 61 (58–65)% (Supplemental Table 4). The survival benefit from CRT-D vs. CRT-P was confirmed by both univariable and multivariable analysis (unadjusted HR 0.82, 95% CI 0.70–0.96, p = 0.013 and adjusted HR 0.78, 95% CI 0.66–0.93, p = 0.005) (Fig. 1C). We also observed that patients with CRT-D exhibited significantly lower mortality than those with CRT-P for up to 9 years after the implantation (Fig. 2C, Supplemental Table 4). In terms of the composite endpoint, CRT-D was associated with better outcomes than CRT-P based on the univariable (unadjusted HR 0.86, 95% CI 0.74–0.99, p = 0.046) and the multivariable analysis as well (adjusted HR 0.81, 95% CI 0.69–0.96, p = 0.013) (Supplemental Fig. 1C).

In addition, we also assessed the association of the device type with all-cause mortality in patients with and without a history of atrial fibrillation (Supplemental Fig. 2). Multivariable Cox regression analysis revealed that CRT-D was associated with better survival than CRT-P only in the latter (adjusted HR 0.77, 95% CI 0.67–0.89, p < 0.001) but not in the former subgroup of patients (adjusted HR 0.91, 95% CI 0.77–1.07, p = 0.260).

### Clinical characteristics and outcomes of the TDA-derived phenogroups

**Fig. 3:**
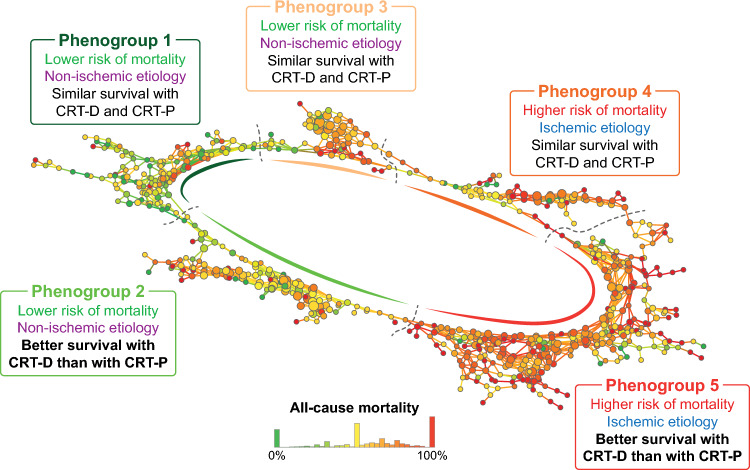
The topological network with the five identified phenogroups of CRT patients. The topological network was created using twenty-five features (metric: normalized correlation, lenses: 2 × multidimensional scaling [resolution: 29, gain: 1.6, equalized]). Each node represents a collection of similar patients, and two nodes are connected if they have at least one patient in common. Nodes are color-coded based on all-cause mortality. The topological network was divided into five regions (i.e., phenogroups) based on all-cause mortality. *CRT* cardiac resynchronization therapy, *CRT-D* cardiac resynchronization therapy defibrillator, *CRT-P* cardiac resynchronization therapy pacemaker

**Fig. 4:**
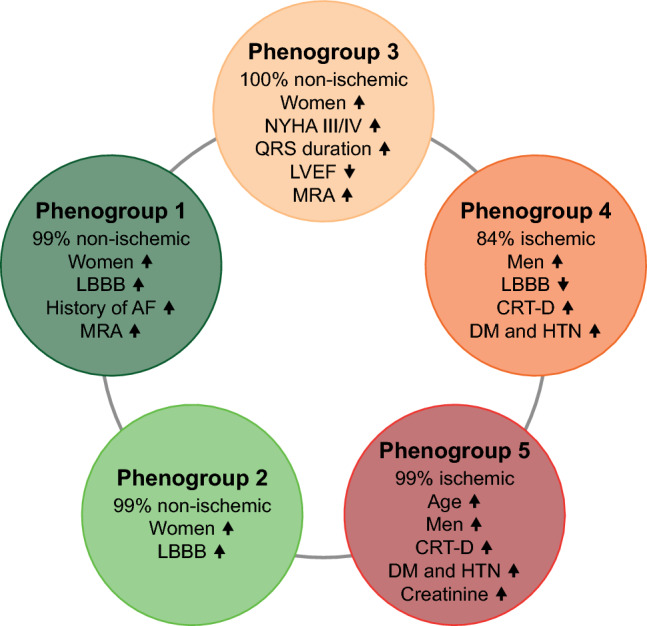
Clinical characteristics of the five phenogroups identified using topological data analysis. *AF* atrial fibrillation, *CRT-D* cardiac resynchronization therapy defibrillator, *DM* diabetes mellitus, *HTN* hypertension, *LBBB* left bundle branch block, *LVEF* left ventricular ejection fraction, *MRA* mineralocorticoid receptor antagonist, *NYHA* New York Heart Association

Using TDA, we created a circular network containing 2359 patients in which we could delineate five phenogroups showing distinct patterns of clinical characteristics and outcomes (Figs. 3 and 4, Table 2, Supplemental Tables 6–10). Etiology appeared to be an important factor, as three phenogroups (1, 2, and 3) included almost exclusively patients with non-ischemic etiology, whereas the other two phenogroups (4 and 5) comprised predominantly ischemic patients (Table 2). As expected, CRT-D was chosen more frequently in the latter two phenogroups than in the non-ischemic ones (Table 2).

**Table 2:** Baseline clinical characteristics of the phenogroups identified using topological data analysis

	Phenogroup 1 n = 321	Phenogroup 2 n = 553	Phenogroup 3 n = 283	Phenogroup 4 n = 254	Phenogroup 5 n = 964
Demographics, physical status, key electrophysiological characteristics					
Age, years	66 (59–74)^‡^	66 (58–73)^‡^	67 (59–73)^‡^	67 (60–75)^‡^	70 (63–76)*^#§†^
Male sex	194 (60)^#†‡^	386 (70)*^†‡^	184 (65)^†‡^	207 (81)*^#§^	809 (84)*^#§^
BMI, kg/m^2^ (1438)	28 (24–31)	28 (25–31)^§^	26 (24–29)^#†‡^	28 (26–30)^§^	27 (24–30)^§^
NYHA III-IV (1981)	150 (57)	298 (57)^§^	106 (66)^#‡^	74 (56)	464 (52)^§^
LBBB	247 (77)^†‡^	426 (77)^§†‡^	197 (70)^#†^	146 (57)*^#§‡^	661 (69)*^#†^
QRS duration, ms (825)	160 (140–180)^§^	160 (140–175)^§^	165 (160–190)*^#‡^	160 (150–176)	160 (140–180)^§^
CRT-D	163 (51)^#§†‡^	226 (41)*^†‡^	115 (41)*^†‡^	173 (68)*^#§^	614 (64)*^#§^
Medical history					
Atrial fibrillation	137 (43)^†‡^	218 (39)^†^	105 (37)	81 (32)*^#^	336 (35)*
Ventricular arrhythmia	75 (23)	120 (22)^‡^	62 (22)	63 (25)	266 (28)^#^
Diabetes mellitus	99 (31)^†‡^	169 (31)^†‡^	98 (35)	102 (40)*^#^	392 (41)*^#^
Hypertension	210 (65)^†‡^	378 (68)^‡^	187 (66)^†‡^	189 (74)*^§^	745 (77)*^#§^
Ischemic etiology	3 (1)^†‡^	6 (1)^†‡^	0 (0)^†‡^	214 (84)*^#§‡^	955 (99)*^#§†^
Myocardial infarction	1 (1)^†‡^	4 (1)^†‡^	0 (0)^†‡^	173 (68)*^#§‡^	764 (79)*^#§†^
PCI	2 (1)^†‡^	1 (1)^†‡^	0 (0)^†‡^	108 (42)*^#§‡^	619 (64)*^#§†^
CABG	1 (0)^†‡^	0 (0)^†‡^	0 (0)^†‡^	60 (24)*^#§‡^	273 (28)*^#§†^
Laboratory measurements					
Creatinine, μmol/L (1527)	91 (77–118)^‡^	96 (78–124)^‡^	83 (70–100)^‡^	92 (77–126)	108 (87–138)*^#§^
BUN, mmol/L (1504)	7 (6–10)^‡^	8 (6–11)^‡^	8 (6–10)	7 (6–10)	9 (7–12)*^#^
Serum sodium, mmol/L (1418)	139 (136–141)	138 (136–140)	140 (140–141)	137 (136–140)	138 (136–140)
Hemoglobin, g/dL (1492)	14 (13–15)^‡^	14 (12–15)	14 (12–15)	14 (14–15)^‡^	14 (12–15)*^†^
Echocardiographic measurements					
LVEDD, mm (1533)	61 (56–68)^#^	64 (59–71)*^‡^	52 (51–56)	65 (58–66)	63 (57–70)^#^
LVESD, mm (1386)	52 (45–58)^#^	54 (47–61)*	43 (42–44)	50 (46–51)	53 (46–60)
LVEF, % (1728)	28 (23–33)^§^	29 (25–33)^§^	25 (22–29)*^#‡^	26 (20–30)	29 (24–34)^§^
Medications					
ACEI/ARB (2171)	274 (92)	485 (92)	212 (95)	176 (90)	847 (91)
Beta-blockers (2169)	278 (93)^†‡^	466 (89)	206 (93)	169 (87)*	825 (89)*
MRA (2169)	225 (76)^#‡^	343 (65)*^§^	167 (75)^#‡^	139 (72)	602 (65)*^§^
Loop diuretics (2170)	230 (77)^†^	407 (78)^†^	183 (82)	167 (86)*^#‡^	735 (79)^†^
Amiodarone (2161)	70 (24)	134 (26)	51 (23)	54 (28)	264 (28)
OAC (2153)	117 (40)^‡^	177 (34)	78 (35)	66 (35)	270 (30)*

The value (in parenthesis) after a feature’s name indicates the number of patients with available data. If there is no value reported, data were available for all patients. Continuous variables are expressed as mean ± standard deviation or median (interquartile range), whereas categorical variables are reported as frequencies (n) and percentages (%). The characteristics of the five phenogroups were compared in a pairwise manner using the Kolmogorov–Smirnov test (for continuous variables) and Chi-squared or Fisher’s exact test (for categorical variables), as appropriate

*BMI* body mass index, *BUN* blood urea nitrogen, *CABG* coronary artery bypass graft surgery, *CRT-D* cardiac resynchronization therapy defibrillator, *CRT-P* cardiac resynchronization therapy pacemaker, *LBBB* left bundle branch block, *LVEDD* left ventricular end-diastolic diameter, *LVEF* left ventricular ejection fraction, *LVESD* left ventricular end-systolic diameter, *MRA* mineralocorticoid receptor antagonist, *NYHA* New York Heart Association, *OAC* oral anticoagulant, *PCI* percutaneous coronary intervention

*p < 0.05 vs. phenogroup 1, ^#^p < 0.05 vs. phenogroup 2, ^§^p < 0.05 vs. phenogroup 3, ^†^p < 0.05 vs. phenogroup 4, ^‡^p < 0.05 vs. phenogroup 5

When we compared the non-ischemic phenogroups (Table 2), we found that patients in phenogroup 1 were more likely to be women (40 vs. 30%, p = 0.006), had smaller LV end-diastolic and end-systolic diameters (61 (56–68) vs. 64 (59–71) mm, p = 0.001 and 52 (45–58) vs. 54 (47–61) mm, p = 0.025, respectively), and a higher percentage of them took MRAs than in phenogroup 2 (76 vs. 65%, p = 0.003). In phenogroup 3, a higher percentage of patients presented with an NYHA functional class III or IV than in phenogroup 2 (66 vs. 57%, p = 0.041). Among the three non-ischemic groups, phenogroup 3 showed the lowest LVEF, the longest QRS duration, and the lowest rate of LBBB (Table 2).

When the two dominantly ischemic phenogroups were compared (Table 2), we observed that patients in phenogroup 5 were older (70 (63–76) vs. 67 (60–75) years, p = 0.009), had lower hemoglobin concentration (14 (12–15) vs. 14 (14–15) g/dL, p = 0.020), presented more frequently with LBBB (69 vs. 57%, p = 0.001), and were more likely to have ischemic etiology than individuals in phenogroup 4 (99 vs. 84%, p < 0.001).

**Fig. 5:**
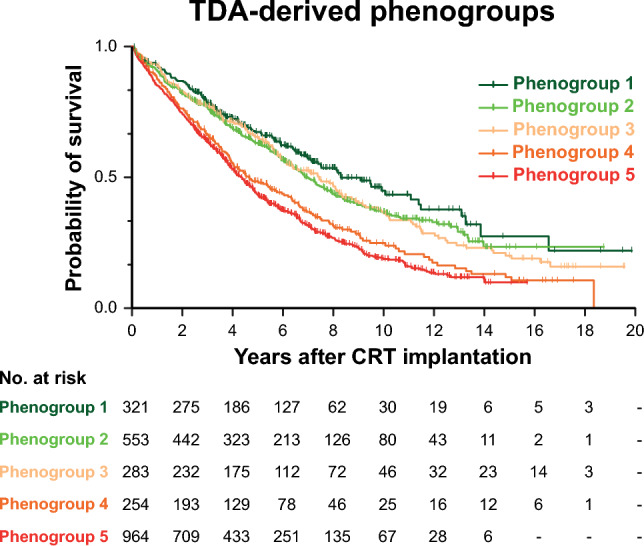
Kaplan–Meier estimates of the time to death from any cause in the five phenogroups identified using topological data analysis. *CRT* cardiac resynchronization therapy, *TDA* topological data analysis

Differences could also be observed in all-cause mortality between the phenogroups (Fig. 5, Supplemental Table 11). Although the survival of the three non-ischemic phenogroup was not significantly different from each other and the two dominantly ischemic phenogroups exhibited similar survival, all three non-ischemic phenogroups showed significantly better survival than those including dominantly ischemic patients (Fig. 5, Supplemental Table 11). In the sensitivity analysis, we also found similar results regarding the composite endpoint (Supplemental Fig. 3, Supplemental Table 12).

### ICD-related survival benefit in the different phenogroups

**Fig. 6:**
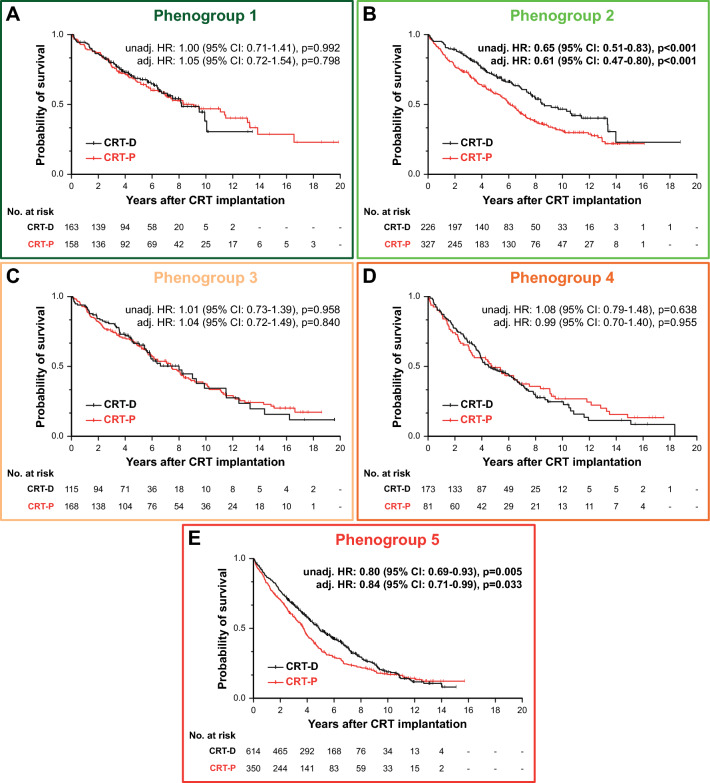
Kaplan–Meier estimates of the time to death from any cause in CRT-D and CRT-P patients in the five phenogroups identified using topological data analysis. Univariable and multivariable Cox proportional hazards models were used to compute hazard ratios with 95% confidence intervals. Each multivariable model included the following features: device type, age, sex, history of atrial fibrillation, and history of ventricular arrhythmia. *CI* confidence interval, *CRT-D* cardiac resynchronization therapy defibrillator, *CRT-P* cardiac resynchronization therapy pacemaker, *HR* hazard ratio

The survival of CRT-D and CRT-P patients were plotted and compared in each phenogroup (Fig. 6). From the three non-ischemic phenogroups, CRT-D was superior to CRT-P only in phenogroup 2 (unadjusted HR 0.65, 95% CI 0.51–0.83, p < 0.001, adjusted HR 0.61, 95% CI 0.47–0.80, p < 0.001) (Fig. 6B). From the two ischemic phenogroups, CRT-D was associated with a significantly lower risk of all-cause mortality compared to CRT-P only in phenogroup 5 (unadjusted HR 0.80, 95% CI 0.69–0.93, p = 0.005, adjusted HR 0.84, 95% CI 0.71–0.99, p = 0.033) (Fig. 6E). In the sensitivity analysis, similar trends could be observed regarding the associations between the device type and the composite of all-cause death, heart transplantation, and left ventricular assist device implantation (Supplemental Fig. 4).

## Discussion

To identify patients with a greater survival benefit from a CRT-D than a CRT-P, we applied TDA, an advanced data analysis approach that is aptly suited for simultaneously evaluating various clinical features. With this technique, we could delineate five distinct phenogroups: three containing almost exclusively non-ischemic and two including predominantly ischemic patients. All three non-ischemic phenogroups showed significantly better survival than those including dominantly ischemic patients. CRT-D was superior to CRT-P in terms of all-cause mortality only in one non-ischemic (phenogroup 2) and one ischemic phenogroup (phenogroup 5). Many of the differences between the characteristics of the phenogroups were subtle but still statistically significant. Importantly, these differences might remain hidden from the physicians during the preimplantation assessment but were efficiently captured by TDA, implying that this approach may have a relevant role in optimizing device selection and improving survival.

The need for an ICD in CRT candidates, especially for primary prevention, is still a subject of debate. The current guidelines recommend a complex, personalized preimplantation risk assessment evaluating several clinical features associated with the risk of SCD and cardiac and non-cardiac (i.e., comorbidity-driven) mortality [12]. Nevertheless, in everyday clinical practice, this assessment is challenging and might lead to incongruent and suboptimal use of the devices.

CRT can reduce per se the risk of SCD by inducing reverse remodeling [13, 14]. In addition, the technological advancements and the emergence of new heart failure medications (such as angiotensin receptor-neprilysin inhibitors and sodium-glucose co-transporter 2 inhibitors) during the last years resulted in a substantial decrease in the incidence of SCD [15, 16]. Moreover, the patient population has changed over the past decades (e.g., also older patients are being considered for CRT implantation) as CRT indications were refined and extended, increasing the proportion of non-cardiac diseases among the underlying causes of death. At the same time, CRT-D is still associated with a higher risk of complications and inappropriate ICD therapies, highlighting the importance and challenges of choosing the optimal device [12].

To this date, the Comparison of Medical Therapy, Pacing, and Defibrillation in Heart Failure (COMPANION) is the only trial that randomized patients to CRT-P or CRT-D [2], although it was designed to compare the effects of CRT with optimal medical therapy and not CRT-D vs. CRT-P. In this trial, it was found that CRT-P was not significantly associated with a reduction in the risk of all-cause mortality, whereas CRT-D was associated with a 36% risk reduction. Nevertheless, these findings and those reported by observational studies are still insufficient to firmly prove or refute the superiority of CRT-D over CRT-P. The ongoing Re-evaluation of Optimal Re-synchronization Therapy in Patients with Chronic Heart Failure (RESET-CRT) trial, hypothesizing that CRT-P is non-inferior to CRT-D for all-cause mortality, is expected to provide crucial information to address this clinically important evidence gap [17]. As a prelude to the RESET-CRT randomized controlled trial, a population-based weighted cohort study including 3569 CRT patients was conducted with the same inclusion and exclusion criteria and primary endpoint [18]. Similar to our results, patients without a defibrillator were significantly older, which might explain the higher rate of all-cause mortality in CRT-P vs. CRT-D patients. However, after adjusting for age and entropy balancing for baseline clinical characteristics, CRT-P was proved to be non-inferior in terms of survival. If the ongoing randomized controlled trial results in the same findings, the clinical relevance of optimal device selection on the overall survival will be confirmed, leaving us with the question of how physicians could be supported to appropriately select those CRT candidates who show an additional survival benefit from an ICD [19].

Per the current guidelines, ischemia and the presence of myocardial fibrosis or scar tissue are the primary factors that should be considered when choosing CRT-D over CRT-P [12]. Multiple studies have reported that ischemic patients have a clear mortality benefit from having an ICD [5, 20–23]. In our patient cohort, CRT-D was found to be superior to CRT-P in terms of all-cause mortality, showing a waning survival benefit over time (Fig. 2). These results may imply that CRT-D reduces SCD predominantly in the first 6–9 years after implantation, but with time, other cardiac or non-cardiac factors (i.e., comorbidities) become the most prevalent causes of death, resulting in decreased benefit from CRT-D vs. CRT-P. A similar trend was also observed by Doran et al. in a post hoc analysis of the COMPANION trial [13]. Nevertheless, they found that CRT-D was associated with better survival than CRT-P in non-ischemic patients but not in ischemic patients. Of note, when the COMPANION trial was conducted, the effect of optimal medical therapy on reverse remodeling or reducing the risk of SCD was not as impressive as nowadays.

In non-ischemic patients, the use of primary prevention ICD is a more challenging question, especially concerning long-term all-cause mortality. The Danish Study to Assess the Efficacy of ICDs in Patients With Nonischemic Systolic Heart Failure on Mortality (DANISH), one of the most recent randomized controlled trials in the field, was designed to investigate the effect of primary-prevention ICD implantation on mortality in patients with non-ischemic heart failure, of whom 58% received CRT as well [24]. Although the risk of SCD was reduced by 50% during the 5-year-long follow-up period, there was no difference in all-cause mortality in the entire study cohort (irrespective of CRT status), only in patients under 68 years of age, which suggests that non-cardiovascular factors are responsible for a higher proportion of death in the elderly [24].

These incongruent findings and the complex nature of the clinical assessment call for advanced data analysis approaches (e.g., machine learning and TDA) to facilitate the identification of those CRT candidates who are most likely to experience an additional mortality benefit from an ICD. To the best of our knowledge, our study is the first that used TDA for this exact purpose. Nevertheless, phenogrouping of heart failure patients has been previously performed by Cikes et al., who applied unsupervised machine learning techniques (multiple kernel learning and k-means clustering) in a subset of MADIT-CRT (Multicenter Automatic Defibrillator Implantation Trial with Cardiac Resynchronization Therapy) patients to integrate clinical features and imaging data in order to identify those who are most likely to respond to CRT [25]. Despite the apparent differences in study design, population, and methods, both their and our study highlighted the relevance of utilizing advanced data analysis techniques for identifying responders to specific therapies. Moreover, similar to their findings, we also noted the accumulation of clinical characteristics known to be predictive of volumetric response (such as female sex, non-ischemic etiology, LBBB, longer QRS duration) in some of the phenogroups (i.e., phenogroups 1, 2, and 3) (Fig. 4).

In our study, TDA could identify one ischemic and one non-ischemic phenogroup that showed mortality benefit from the implantation of a CRT-D instead of a CRT-P. These results confirm that CRT candidates form a heterogenous population of heart failure patients, and several distinct phenotypes exist within both the ischemic and non-ischemic patient subsets. Our study also showcases the capabilities of TDA to capture the subtle differences in the characteristics of these patients, suggesting that this approach may have a relevant role in optimizing device selection and improving outcomes.

### Limitations

Besides its strength, our study has several limitations that should be acknowledged. First, the large dataset we analyzed in this study was derived from a single center. Thus, additional investigations must be conducted in the future to confirm our findings in external cohorts. Second, due to the retrospective nature of data collection, the proportion of missing values was relatively high in our dataset; hence we had to omit several well-established prognostic markers (e.g., N-terminal pro-brain natriuretic peptide) from TDA. Third, a CRT-D or a CRT-P device was implanted based on the physicians’ clinical judgment and not in a randomized fashion, which may have resulted in selection bias. Fourth, the primary endpoint of our study was all-cause mortality, as cause-specific mortality data was unavailable. Moreover, we could not investigate reverse remodeling either, as follow-up echocardiographic data were available only for a limited number of patients (< 10%) in our dataset. Fifth, despite the lack of large, randomized trials proving the usefulness of CRT in patients with permanent atrial fibrillation, we did not limit our analysis to patients in sinus rhythm as we wanted to explore the data of all patients who underwent successful CRT implantation at our center. Nevertheless, the subgroup analysis revealed that CRT-D was associated with better survival than CRT-P only in patients with no history of atrial fibrillation. Last, the generated topological network cannot be used directly to classify new patients. Nonetheless, patients in the network can be labeled based on their location, and then, using this labeled data, machine learning classifiers can be trained to allocate new patients to the identified phenogroups. However, as we did not have access to any external datasets required for the thorough validation of such classifiers, we decided to postpone their development and validation until additional datasets become available.

## Conclusions

In this retrospective observational study, CRT-D was found to be superior in reducing all-cause mortality compared to CRT-P in the entire cohort and both ischemic and non-ischemic patient subgroups. By simultaneously evaluating various clinical features, TDA could identify distinct phenogroups even within ischemic and non-ischemic subsets of CRT candidates and be able to pinpoint those who are more likely to show additional benefit from the implantation of a CRT-D instead of a CRT-P in terms of all-cause mortality.

## Supplementary Information

Below is the link to the electronic supplementary material.

Supplementary file1 (PDF 2216 KB)

## Data Availability

The data supporting the findings of this study are available from the corresponding author upon reasonable request.
