# Supplementary material for: Topological data analysis to identify cardiac resynchronization therapy patients exhibiting benefit from an implantable cardioverter-defibrillator
Source: Clin Res Cardiol. 2023 Aug 25;113(10):1430–42. doi: 10.1007/s00392-023-02281-6 (PMC11420357; doi:10.1007/s00392-023-02281-6)
Supplement: Supplementary file 1 — Supplementary file1 (PDF 2216 KB) [file 392_2023_2281_MOESM1_ESM.pdf]

SUPPLEMENTARY INFORMATION

Topological data analysis to identify cardiac resynchronization therapy patients exhibiting benefit from an implantable cardioverter-defibrillator

Supplemental Table 1 Feature selection

	Demographics, clinical characteristics, and CV risk factors	ECG	Echocardiography	Laboratory results	Medications
**Features used in topological data analysis**	Age<br>Sex<br>BMI<br>NYHA functional class<br>Etiology of heart failure<br>Hypertension<br>Diabetes<br>History of AF<br>History of VA<br>History of MI<br>History of CABG<br>History of PCI<br>CRT-D or CRT-P	QRS morphology	LVEDD<br>LVESD<br>LVEF	Hemoglobin<br>Serum sodium<br>Serum creatinine<br>BUN	Beta-blockers<br>MRA<br>Amiodarone<br>OAC
**Features excluded due to ≥40% missing values**	Systolic blood pressure<br>Diastolic blood pressure	Pre- and postimplant QRS duration<br>PR interval	IVSd<br>LVPWd<br>LVEDV<br>LVESV<br>TAPSE	Lymphocytes<br>Total cholesterol<br>Serum uric acid<br>NT-proBNP	

AF – atrial fibrillation, BMI – body mass index, BUN – blood urea nitrogen, CABG – coronary artery bypass grafting, CRT-D – cardiac resynchronization therapy defibrillator, CRT-P – cardiac resynchronization therapy pacemaker, CV – cardiovascular, ECG – electrocardiogram, GFR – glomerular filtration rate, IVSd – thickness of the interventricular septum at end-diastole, LVEDD – left ventricular end-diastolic diameter, LVEDV – left ventricular end-diastolic volume, LVEF – left ventricular ejection fraction, LVESD – left ventricular end-systolic diameter, LVESV – left ventricular end-systolic volume, LVPWd – thickness of the left ventricular posterior wall at end-diastole, MI – myocardial infarction, MRA – mineralocorticoid receptor antagonists, NYHA – New York Heart Association, NT-proBNP – N-terminal pro-brain natriuretic peptide, OAC – oral anticoagulants, PCI – percutaneous coronary intervention, TAPSE – tricuspid annular plane systolic excursion, VA – ventricular arrhythmia

Supplemental Table 2 Baseline clinical characteristics of patients with ischemic etiology

	All<br>n=1,273	CRT-P<br>n=456	CRT-D<br>n=817	P-value
**Demographics, physical status, key electrophysiological characteristics**				
Age, years	70 (63-76)	72 (64-78)	69 (62-74)	<0.001
Male sex	1,039 (82)	335 (74)	704 (86)	<0.001
BMI, kg/m^2 (832)	27 (25-30)	27 (24-30)	28 (25-31)	0.211
NYHA III-IV (1,108)	596 (54)	246 (61)	354 (50)	<0.001
LBBB	861 (68)	318 (70)	543 (66)	0.236
QRS duration, ms (455)	160 (140-180)	160 (150-186)	160 (140-177)	0.006
**Medical history**				
Atrial fibrillation	480 (38)	189 (42)	291 (36)	0.041
Ventricular arrhythmia	358 (28)	55 (12)	303 (37)	<0.001
Diabetes mellitus	538 (42)	194 (43)	344 (42)	0.906
Hypertension	997 (78)	365 (80)	632 (77)	0.288
Myocardial infarction	998 (78)	354 (78)	644 (79)	0.620
PCI	771 (61)	265 (58)	506 (62)	0.189
CABG	349 (27)	99 (22)	250 (31)	<0.001
**Laboratory measurements**				
NT-proBNP, pg/mL(187)	2,610<br>(1,290-3,713)	2,758<br>(1,410-4,516)	2,587<br>(1,235-3,671)	0.368
Creatinine, μmol/L (899)	107 (86-137)	108 (84-138)	107 (88-135)	0.963
eGFR, mL/min/1.73m^2 (899)	61 (46-77)	58 (44-78)	62 (47-77)	0.234
BUN, mmol/L (890)	9 (7-12)	9 (7-13)	9 (6-12)	0.099
Serum sodium, mmol/L (840)	138 (136-140)	138 (136-141)	138 (136-140)	0.234
Hemoglobin, g/dL (880)	13±2	14±2	13±2	0.987
**Echocardiographic measurements**				
LVEDD, mm (881)	63±10	62±10	64±9	0.002
LVESD, mm (786)	53±10	52±10	54±10	0.001
LVEF, % (977)	29 (24-34)	30 (25-35)	28(24-33)	<0.001
**Medications**				
ACEI/ARB (1,199)	1,093 (91)	372 (89)	721 (92)	0.088
Beta-blockers (1,199)	1,067 (89)	368 (88)	699 (89)	0.562
MRA (1,197)	806 (67)	254 (61)	552 (71)	<0.001
Loop diuretics (1,199)	965 (81)	343 (82)	622 (80)	0.284
Amiodarone (1,196)	352 (29)	96 (23)	256 (33)	<0.001
OAC (1,181)	341 (29)	124 (30)	217 (28)	0.545

The value (in parenthesis) after a feature's name indicates the number of patients with available data. If there is no value reported, data were available for all patients. Continuous variables are expressed as mean ± standard deviation or median (interquartile range), whereas categorical variables are reported as frequencies (n) and percentages (%). The characteristics of the CRT-P and CRT-D groups were compared using unpaired Student's t-test or Mann-Whitney U test for continuous variables and Chi-squared or Fisher's exact test for categorical variables, as appropriate.

ACEI – angiotensin-converting enzyme inhibitor, ARB – angiotensin receptor blocker, BMI – body mass index, eGFR – estimated glomerular filtration rate, LBBB – left bundle branch block, other abbreviations as in Supplemental Table 1.

Supplemental Table 3 Baseline clinical characteristics of patients with non-ischemic etiology

	All n=1,330	CRT-P n=733	CRT-D n=597	P-value
**Demographics, physical status, key electrophysiological characteristics**				
Age, years	66 (59-73)	68 (60-74)	64 (57-71)	<0.001
Male sex	908 (68)	463 (63)	445 (75)	<0.001
BMI, kg/m^2 (751)	28 (25-31)	27 (24-31)	28 (25-31)	0.718
NYHA III-IV (1,067)	613 (58)	365 (62)	248 (52)	0.002
LBBB	961 (72)	523 (71)	438 (73)	0.424
QRS duration, ms (455)	160 (140-178)	160 (140-180)	160 (140-170)	0.081
**Medical history**				
Atrial fibrillation	504 (38)	282 (39)	222 (37)	0.650
Ventricular arrhythmia	296 (22)	75 (10)	221 (37)	<0.001
Diabetes mellitus	412 (31)	237 (32)	175 (29)	0.257
Hypertension	880 (66)	493 (67)	387 (65)	0.352
**Laboratory measurements**				
NT-proBNP, pg/mL(162)	2,934 (1,631-4,256)	2,882 (1,642-3,417)	2,968 (1,603-4,686)	0.680
Creatinine, µmol/L (782)	94 (77-123)	93 (78-126)	96 (77-119)	0.508
eGFR, mL/min/1.73m^2 (783)	68 (50-85)	65 (48-84)	69 (53-85)	0.013
BUN, mmol/L (768)	8 (6-11)	8 (6-11)	8 (6-11)	0.330
Serum sodium, mmol/L (723)	139 (136-141)	139 (136-141)	139 (136-140)	0.588
Hemoglobin, g/dL (766)	14±2	14±2	14±2	0.755
**Echocardiographic measurements**				
LVEDD, mm (798)	63 (58-70)	63 (57-70)	64 (58-70)	0.113
LVESD, mm (714)	54 (47-61)	53 (46-60)	55 (49-62)	0.003
LVEF, % (926)	28 (24-32)	29 (25-34)	27 (23-30)	<0.001
**Medications**				
ACEI/ARB (1,181)	1,093 (93)	581 (92)	512 (94)	0.222
Beta-blockers (1,178)	1,048 (89)	547 (87)	501 (92)	0.005
MRA (1,180)	808 (69)	399 (63)	409 (75)	<0.001
Loop diuretics (1,180)	923 (78)	489 (77)	434 (79)	0.397
Amiodarone (1,172)	289 (25)	119 (19)	170 (31)	<0.001
OAC (1,174)	362 (31)	204 (32)	158 (29)	0.229

The value (in parenthesis) after a feature's name indicates the number of patients with available data. If there is no value reported, data were available for all patients. Continuous variables are expressed as mean ± standard deviation or median (interquartile range), whereas categorical variables are reported as frequencies (n) and percentages (%). The characteristics of the CRT-P and CRT-D groups were compared using unpaired Student's t-test or Mann-Whitney U test for continuous variables and Chi-squared or Fisher's exact test for categorical variables, as appropriate.

Abbreviations as in Supplemental Tables 1 and 2.

Supplemental Table 4 Mortality and absolute risk reduction calculated based on Kaplan-Meier estimates

		Years after CRT implantation									
		1	2	3	4	5	6	7	8	9	10
**All patients**											
**Mortality, %**	**All**	11.3 (10.1-12.6)	20.7 (19.1-22.2)	28.9 (27.1-30.7)	37.8 (35.8-39.6)	45.2 (43.1-47.1)	51.1 (49.0-53.1)	57.3 (55.1-59.4)	62.4 (60.2-64.5)	66.5 (64.2-68.6)	70.5 (68.1-72.7)
	**CRT-D**	10.3 (8.7-11.9)	19.2 (17.1-21.2)	27.0 (24.6-29.3)	36.0 (33.3-38.5)	43.2 (40.4-45.8)	48.6 (45.6-51.3)	55.5 (52.4-58.5)	61.9 (58.6-65.0)	66.2 (62.8-69.4)	70.9 (67.3-74.1)
	**CRT-P**	12.6 (10.7-14.5)	22.4 (20.0-24.8)	31.2 (28.5-33.8)	39.9 (37.0-42.7)	47.5 (44.5-50.4)	54.0 (50.9-56.9)	59.5 (56.3-62.4)	63.5 (60.3-66.4)	67.3 (64.1-70.2)	70.8 (67.6-73.7)
**ARR** (CRT-D vs. CRT-P), pp		2.3 (-0.2-4.8)	**3.3 (0.2-6.4)**	**4.3 (0.8-7.8)**	**3.9 (0.0-7.8)**	**4.4 (0.3-8.5)**	**5.4 (1.3-9.5)**	3.9 (-0.4-8.2)	1.6 (-2.7-5.9)	1.1 (-3.4-5.6)	-0.1 (-4.6-4.4)
**Ischemic patients**											
**Mortality, %**	**All**	14.4 (12.4-16.3)	24.7 (22.2-27.0)	34.7 (32.0-37.3)	45.2 (42.3-47.9)	54.3 (51.3-57.1)	60.1 (57.1-62.9)	67.0 (64.0-69.7)	71.9 (68.9-74.7)	75.4 (72.4-78.1)	79.6 (76.5-82.2)
	**CRT-D**	13.2 (10.8-15.5)	22.4 (19.5-25.2)	31.5 (28.2-34.6)	41.6 (38-45.0)	50.7 (47.0-54.2)	56.1 (52.3-59.6)	63.5 (59.5-67.1)	70.7 (66.6-74.3)	75.2 (71.0-78.8)	79.6 (75.3-83.2)
	**CRT-P**	16.5 (13.0-19.8)	28.6 (24.3-32.7)	40.3 (35.6-44.7)	51.5 (46.6-56.0)	60.5 (55.6-64.9)	66.9 (62.0-71.2)	72.8 (68.1-76.9)	74.9 (70.2-78.8)	77.1 (72.4-80.9)	80.8 (76.3-84.5)
**ARR** (CRT-D vs. CRT-P), pp		3.3 (-0.8-7.4)	**6.2 (1.1-11.3)**	**8.8 (3.3-14.3)**	**9.9 (4.0-15.8)**	**9.8 (3.9-15.7)**	**10.8 (4.9-16.7)**	**9.3 (3.4-15.2)**	4.2 (-1.5-9.9)	1.9 (-3.8-7.6)	1.2 (-4.5-6.9)
**Non-ischemic patients**											
**Mortality, %**	**All**	8.5 (6.9-9.9)	16.8 (14.8-18.8)	23.4 (21.1-25.7)	30.6 (28.0-33.1)	36.3 (33.5-38.9)	42.3 (39.4-45.1)	47.8 (44.7-50.8)	53.0 (49.7-56.1)	57.6 (54.2-60.8)	61.4 (57.8-64.7)
	**CRT-D**	6.4 (4.4-8.3)	14.7 (11.8-17.5)	20.8 (17.4-24.0)	28.2 (24.4-31.9)	32.5 (28.4-36.3)	37.9 (33.4-42.0)	43.8 (38.9-48.3)	48.6 (43.2-53.5)	52.4 (46.6-57.5)	57.3 (51.0-62.8)
	**CRT-P**	10.2 (7.9-12.3)	18.6 (15.7-21.4)	25.5 (22.3-28.7)	32.5 (29.0-35.9)	39.3 (35.5-42.8)	45.7 (41.7-49.4)	50.9 (46.8-54.7)	56.2 (52.0-60.0)	61.1 (56.8-65.0)	64.2 (59.8-68.2)
**ARR** (CRT-D vs. CRT-P), pp		**3.8 (0.9-6.7)**	**3.9 (0.0-7.8)**	**4.8 (0.3-9.3)**	4.3 (-0.8-9.4)	**6.8 (1.3-12.3)**	**7.9 (2.2-13.6)**	**7.1 (1.0-13.2)**	**7.6 (1.1-14.1)**	**8.8 (1.9-15.7)**	6.9 (-0.4-14.2)

Supplemental Table 4 Continued

		Years after CRT implantation									
		…	11	12	13	14	15	16	17	18	19
**All patients**											
**Mortality, %**	**All**	…	73.4 (70.9-75.6)	76.6 (74.1-78.9)	79.4 (76.8-81.7)	82.3 (79.6-84.7)	84.7 (81.6-87.2)	85.5 (82.3-88.0)	87.3 (83.7-90.1)	87.3 (83.7-90.1)	89.1 (83.9-92.7)
	**CRT-D**	…	75.8 (72.0-79.2)	79.2 (75.2-82.6)	80.9 (76.8-84.3)	83.8 (79.1-87.4)	86.5 (81.2-90.3)	87.9 (82.1-91.8)	89.6 (83.0-93.7)	89.6 (83.0-93.7)	92.2 (83.5-96.3)
	**CRT-P**	…	72.2 (69.0-75.1)	75.4 (72.1-78.3)	78.8 (75.4-81.7)	81.8 (78.2-84.8)	83.8 (79.9-87.0)	84.4 (80.4-87.6)	86.4 (81.7-89.8)	86.4 (81.7-89.8)	86.4 (81.7-89.8)
**ARR** (CRT-D vs. CRT-P), pp		…	-3.6 (-8.3-1.1)	-3.8 (-8.7-1.1)	-2.1 (-7.0-2.8)	-2.0 (-7.3-3.3)	-2.7 (-8.4-3.0)	-3.5 (-9.4-2.4)	-3.3 (-9.8-3.2)	-3.3 (-9.8-3.2)	-5.9 (-13.0-1.2)
**Ischemic patients**											
**Mortality, %**	**All**	…	82.6 (79.5-85.2)	85.4 (82.2-88.0)	87.6 (84.3-90.2)	89.3 (85.7-92.0)	92.0 (87.5-94.9)	93.2 (88.3-96.0)	93.2 (88.3-96.0)	93.2 (88.3-96.0)	-
	**CRT-D**	…	84.7 (80.2-88.1)	88.3 (83.6-91.6)	89.1 (84.3-92.4)	89.1 (84.3-92.4)	90.9 (84.8-94.5)	95.4 (80.0-99.0)	95.4 (80.0-99.0)	95.4 (80.0-99.0)	-
	**CRT-P**	…	81.7 (77.1-85.3)	83.6 (79.0-87.2)	87.0 (82.1-90.6)	89.9 (84.4-93.5)	92.8 (86.4-96.2)	92.8 (86.4-96.2)	92.8 (86.4-96.2)	-	-
**ARR** (CRT-D vs. CRT-P), pp		…	-3.0 (-8.7-2.7)	-4.6 (-10.3-1.1)	-2.0 (-7.7-3.7)	0.9 (-5.0-6.8)	1.9 (-4.6-8.4)	-2.6 (-10.8-5.6)	-2.6 (-10.8-5.6)	-	-
**Non-ischemic patients**											
**Mortality, %**	**All**	…	64.1 (60.4-67.4)	67.8 (63.9-71.2)	71.2 (67.2-74.8)	75.4 (71.0-79.1)	77.8 (73.1-81.7)	78.5 (73.7-82.4)	81.7 (75.9-86.1)	81.7 (75.9-86.1)	81.7 (75.9-86.1)
	**CRT-D**	…	61.9 (55.1-67.7)	64.6 (57.4-70.6)	67.8 (60.1-74.0)	74.5 (65-81.4)	78.7 (68.2-85.8)	78.7 (68.2-85.8)	82.3 (69.6-89.7)	82.3 (69.6-89.7)	82.3 (69.6-89.7)
	**CRT-P**	…	66.1 (61.6-70.0)	70.0 (65.4-74.1)	73.4 (68.6-77.5)	76.6 (71.5-80.7)	78.2 (72.9-82.5)	79.2 (73.6-83.6)	82.2 (75.5-87.0)	82.2 (75.5-87.0)	82.2 (75.5-87.0)
**ARR** (CRT-D vs. CRT-P), pp		…	4.2 (-3.4-11.8)	5.4 (-2.4-13.2)	5.6 (-2.6-13.8)	2.1 (-7.1-11.3)	-0.5 (-10.3-9.3)	0.5 (-9.5-10.5)	-0.1 (-11.3-11.1)	-0.1 (-11.3-11.1)	-0.1 (-11.3-11.1)

Mortality and absolute risk reduction are reported with 95% confidence intervals. Values in bold indicate significant absolute risk reduction (CRT-D vs. CRT-P). Absolute risk reduction was considered significant if the value 0 fell outside of its confidence interval.

ARR – absolute risk reduction, CRT – cardiac resynchronization therapy, CRT-D – cardiac resynchronization therapy defibrillator, CRT-P – pacemaker, pp – percentage point

Supplemental Table 5 Sequential Cox regression models for the prediction of all-cause mortality

	Model 1<br>AIC: 22,435		Model 2<br>AIC: 22,170		Model 3<br>AIC: 22,166		Model 4<br>AIC: 22,113	
	**HR (95% CI)**	**P-value**	**HR (95% CI)**	**P-value**	**HR (95% CI)**	**P-value**	**HR (95% CI)**	**P-value**
CRT-D	0.94 (0.85-1.03)	0.187	0.94 (0.85-1.04)	0.218	0.90 (0.81-1.00)	0.052	0.83 (0.74-0.92)	<0.001
Age, years			1.03 (1.03-1.04)	<0.001	1.03 (1.03-1.04)	<0.001	1.03 (1.02-1.03)	<0.001
Male sex			1.71 (1.51-1.93)	<0.001	1.69 (1.49-1.91)	<0.001	1.60 (1.41-1.81)	<0.001
History of AF			1.31 (1.18-1.45)	<0.001	1.30 (1.18-1.44)	<0.001	1.32 (1.20-1.46)	<0.001
Ventricular arrhythmia					1.16 (1.03-1.30)	0.014	1.16 (1.03-1.30)	0.015
Ischemic etiology							1.49 (1.34-1.65)	<0.001

AF – atrial fibrillation, AIC – Akaike information criterion, CI – confidence interval, CRT-D – cardiac resynchronization therapy defibrillator, HR – hazard ratio

Supplemental Table 6 Baseline clinical characteristics of the first phenogroup

	All patients n=321	CRT-P n=158	CRT-D n=163	P-value
**Demographics, physical status, key electrophysiological characteristics**				
Age, years	66 (59-74)	69 (62-76)	63 (57-71)	0.002
Male sex	194 (60)	82 (52)	112 (69)	0.003
BMI, kg/m^2 (182)	28 (24-31)	28 (24-31)	27 (24-31)	0.304
NYHA III-IV (264)	150 (57)	77 (61)	73 (53)	0.485
LBBB	247 (77)	116 (73)	131 (80)	0.147
**Medical history**				
Atrial fibrillation	137 (43)	72 (46)	65 (40)	0.312
Ventricular arrhythmia	74 (23)	15 (10)	60 (37)	<0.001
Diabetes mellitus	99 (31)	52 (33)	47 (29)	0.469
Hypertension	210 (65)	110 (70)	100 (61)	0.128
Ischemic etiology	3 (1)	1 (1)	2 (1)	1.000
Myocardial infarction	1 (1)	0 (0)	1 (1)	1.000
PCI	2 (1)	1 (1)	1 (1)	1.000
CABG	0 (0)	0 (0)	0 (0)	1.000
**Laboratory measurements**				
Creatinine, µmol/L (161)	96 (78-124)	96 (79-132)	96 (77-119)	0.230
BUN, mmol/L (155)	7 (6-10)	8 (6-11)	7 (6-9)	0.041
Serum sodium, mmol/L (142)	139 (136-141)	139 (136-142)	139 (136-140)	0.480
Hemoglobin, g/dL (161)	14 (13-15)	14 (13-15)	14 (13-15)	0.987
**Echocardiographic measurements**				
LVEDD, mm (172)	61 (56-68)	60 (56-65)	63 (56-69)	0.192
LVESD, mm (148)	52 (45-59)	50 (43-56)	53 (47-61)	0.118
LVEF, % (240)	28 (22-33)	28 (25-35)	26 (20-31)	0.053
**Medications**				
Beta-blockers (278)	278 (93)	134 (93)	144 (93)	0.821
MRA (298)	225 (78)	96 (67)	129 (89)	<0.001
Amiodarone (298)	70 (23)	23 (16)	47 (30)	0.004
OAC (298)	119 (40)	58 (41)	61 (40)	0.906

The value (in parenthesis) after a feature's name indicates the number of patients with available data. If there is no value reported, data were available for all patients. Continuous variables are expressed as mean ± standard deviation or median (interquartile range), whereas categorical variables are reported as frequencies (n) and percentages (%). The characteristics of the CRT-P and CRT-D groups were compared using unpaired Student's t-test or Mann-Whitney U test for continuous variables and Chi-squared or Fisher's exact test for categorical variables, as appropriate.

Abbreviations as in Supplemental Tables 1 and 2.

Supplemental Table 7 Baseline clinical characteristics of the second phenogroup

	All patients n=553	CRT-P n=327	CRT-D n=226	P-value
**Demographics, physical status, key electrophysiological characteristics**				
Age, years	66 (58-73)	68 (61-74)	64 (57-70)	0.002
Male sex	386 (70)	214 (65)	172 (76)	0.008
BMI, kg/m^2 (401)	28 (25-31)	28 (25-31)	28 (25-32)	0.530
NYHA III-IV (525)	298 (57)	189 (60)	109 (52)	0.059
LBBB	426 (77)	253 (77)	173 (77)	0.838
**Medical history**				
Atrial fibrillation	218 (39)	136 (42)	82 (36)	0.455
Ventricular arrhythmia	120 (22)	38 (12)	82 (36)	<0.001
Diabetes mellitus	169 (31)	112 (34)	57 (25)	0.025
Hypertension	378 (68)	232 (71)	146 (65)	0.137
Ischemic etiology	6 (1)	5 (2)	1 (1)	0.409
Myocardial infarction	4 (1)	3 (1)	1 (1)	0.648
PCI	1 (1)	1 (1)	0 (0)	1.000
CABG	1 (1)	1 (1)	0 (0)	1.000
**Laboratory measurements**				
Creatinine, µmol/L (514)	96 (78-124)	96 (79-132)	96 (77-119)	0.230
BUN, mmol/L (507)	8 (6-11)	8 (7-11)	8 (6-10)	0.186
Serum sodium, mmol/L (490)	138 (136-140)	138 (136-140)	138 (136-140)	0.980
Hemoglobin, g/dL (494)	14 (13-15)	14 (13-15)	14 (13-15)	0.980
**Echocardiographic measurements**				
LVEDD, mm (550)	64 (59-71)	63 (58-71)	65 (60-71)	0.158
LVESD, mm (517)	54 (47-61)	54 (47-61)	56 (50-63)	0.026
LVEF, % (546)	29 (25-33)	29 (25-34)	28 (24-30)	0.028
**Medications**				
Beta-blockers (524)	466 (89)	270 (88)	196 (91)	0.322
MRA (525)	343 (65)	188 (61)	155 (72)	0.012
Amiodarone (520)	134 (26)	68 (22)	66 (31)	0.033
OAC (525)	178 (34)	109 (35)	69 (32)	0.401

The value (in parenthesis) after a feature's name indicates the number of patients with available data. If there is no value reported, data were available for all patients. Continuous variables are expressed as mean ± standard deviation or median (interquartile range), whereas categorical variables are reported as frequencies (n) and percentages (%). The characteristics of the CRT-P and CRT-D groups were compared using unpaired Student's t-test or Mann-Whitney U test for continuous variables and Chi-squared or Fisher's exact test for categorical variables, as appropriate.

Abbreviations as in Supplemental Tables 1 and 2.

Supplemental Table 8 Baseline clinical characteristics of the third phenogroup

	All patients n=283	CRT-P n=168	CRT-D n=115	P-value
**Demographics, physical status, key electrophysiological characteristics**				
Age, years	67 (59-73)	67 (59-74)	65 (59-71)	0.296
Male sex	184 (65)	104 (62)	80 (70)	0.206
BMI, kg/m^2 (87)	26 (23-29)	26 (24-30)	26 (23-29)	0.965
NYHA III-IV (160)	106 (66)	74 (73)	32 (54)	0.016
LBBB	197 (70)	114 (68)	83 (72)	0.511
**Medical history**				
Atrial fibrillation	105 (37)	57 (34)	48 (42)	0.211
Ventricular arrhythmia	62 (22)	15 (9)	47 (41)	<0.001
Diabetes mellitus	98 (35)	56 (33)	42 (37)	0.612
Hypertension	187 (66)	110 (65)	77 (67)	0.967
Ischemic etiology	0 (0)	0 (0)	0 (0)	1.000
Myocardial infarction	0 (0)	0 (0)	0 (0)	1.000
PCI	0 (0)	0 (0)	0 (0)	1.000
CABG	0 (0)	0 (0)	0 (0)	1.000
**Laboratory measurements**				
Creatinine, µmol/L (19)	83 (69-102)	84 (69-102)	80 (68-103)	0.927
BUN, mmol/L (18)	8 (6-11)	6 (6-9)	8 (7-11)	0.244
Serum sodium, mmol/L (7)	140 (139-142)	140 (140-140)	140 (139-142)	0.905
Hemoglobin, g/dL (24)	14 (12-15)	14 (12-16)	14 (12-15)	0.952
**Echocardiographic measurements**				
LVEDD, mm (4)	52 (50-65)	50 (50-50)	52 (51-69)	-
LVESD, mm (2)	43 (42-44)	-	43 (42-44)	-
LVEF, % (45)	25 (22-30)	27 (20-30)	25 (22-29)	0.868
**Medications**				
Beta-blockers (222)	206 (93)	111 (90)	95 (97)	0.038
MRA (223)	167 (75)	85 (68)	82 (84)	0.008
Amiodarone (221)	51 (23)	20 (16)	31 (32)	0.010
OAC (223)	78 (35)	41 (33)	37 (37)	0.572

The value (in parenthesis) after a feature's name indicates the number of patients with available data. If there is no value reported, data were available for all patients. Continuous variables are expressed as mean ± standard deviation or median (interquartile range), whereas categorical variables are reported as frequencies (n) and percentages (%). The characteristics of the CRT-P and CRT-D groups were compared using unpaired Student's t-test or Mann-Whitney U test for continuous variables and Chi-squared or Fisher's exact test for categorical variables, as appropriate.

Abbreviations as in Supplemental Tables 1 and 2.

Supplemental Table 9 Baseline clinical characteristics of the fourth phenogroup

	All patients n=254	CRT-P n=81	CRT-D n=173	P-value
**Demographics, physical status, key electrophysiological characteristics**				
Age, years	67 (60-75)	68 (59-76)	67 (60-74)	0.411
Male sex	207 (81)	58 (72)	149 (86)	0.009
BMI, kg/m^2 (81)	28 (25-30	27 (25-35)	28 (26-30)	0.992
NYHA III-IV (133)	74 (29)	28 (68)	46 (50)	0.146
LBBB	146 (57)	41 (51)	105 (61)	0.137
**Medical history**				
Atrial fibrillation	61 (24)	28 (35)	53 (31)	0.565
Ventricular arrhythmia	63 (25)	5 (6)	58 (34)	<0.001
Diabetes mellitus	102 (40)	30 (37)	72 (42)	0.583
Hypertension	189 (74)	58 (72)	131 (76)	0.538
Ischemic etiology	214 (84)	63 (78)	151 (87)	0.065
Myocardial infarction	173 (68)	54 (67)	119 (68)	0.776
PCI	108 (43)	29 (36)	79 (46)	0.173
CABG	60 (24)	11 (14)	49 (28)	0.011
**Laboratory measurements**				
Creatinine, µmol/L (20)	92 (76-134)	85 (75-95)	98 (82-142)	0.475
BUN, mmol/L (19)	7 (6-11)	8 (5-11)	7 (6-12)	0.961
Serum sodium, mmol/L (15)	137 (136-141)	139 (137-143)	137 (135-139)	0.461
Hemoglobin, g/dL (25)	14±1	14±1	14±1	0.292
**Echocardiographic measurements**				
LVEDD, mm (11)	63 (57-70)	67 (66-68)	60 (56-66)	0.218
LVESD, mm (8)	50 (45-54)	58 (58-58)	49 (44-50)	-
LVEF, % (38)	26 (20-30)	32 (20-36)	25 (20-30)	0.447
**Medications**				
Beta-blockers (195)	169 (87)	38 (72)	131 (93)	<0.001
MRA (194)	139 (72)	30 (57)	109 (77)	0.007
Amiodarone (195)	54 (28)	13 (25)	41 (29)	0.594
OAC (191)	66 (35)	19 (37)	47 (34)	0.735

The value (in parenthesis) after a feature's name indicates the number of patients with available data. If there is no value reported, data were available for all patients. Continuous variables are expressed as mean ± standard deviation or median (interquartile range), whereas categorical variables are reported as frequencies (n) and percentages (%). The characteristics of the CRT-P and CRT-D groups were compared using unpaired Student's t-test or Mann-Whitney U test for continuous variables and Chi-squared or Fisher's exact test for categorical variables, as appropriate.

Abbreviations as in Supplemental Tables 1 and 2.

Supplemental Table 10 Baseline clinical characteristics of the fifth phenogroup

	All n=964	CRT-P n=350	CRT-D n=614	P-value
**Demographics, physical status, key electrophysiological characteristics**				
Age, years	70 (63-76)	72 (65-79)	69 (62-74)	<0.001
Male sex	809 (84)	273 (78)	536 (87)	<0.001
BMI, kg/m^2 (687)	27 (25-31)	27 (24-30)	28 (25-31)	0.152
NYHA III-IV (899)	464 (52)	185 (57)	279 (49)	0.022
LBBB	661 (69)	248 (71)	413 (67)	0.279
**Medical history**				
Atrial fibrillation	336 (35)	130 (37)	206 (34)	0.262
Ventricular arrhythmia	266 (28)	44 (13)	222 (36)	<0.001
Diabetes mellitus	392 (41)	141 (40)	251 (41)	0.892
Hypertension	745 (77)	276 (79)	469 (76)	0.424
Ischemic etiology	955 (99)	347 (99)	608 (99)	0.983
Myocardial infarction	764 (79)	275 (79)	489 (80)	0.741
PCI	619 (64)	221 (63)	398 (65)	0.872
CABG	273 (28)	80 (23)	193 (31)	0.005
**Laboratory measurements**				
Creatinine, µmol/L (813)	108 (87-138)	110 (85-139)	107 (88-137)	0.603
BUN, mmol/L (805)	9 (7-12)	9 (7-14)	9 (6-12)	0.093
Serum sodium, mmol/L (764)	138 (136-141)	139 (136-141)	138 (135-140)	0.303
Hemoglobin, g/dL (788)	13±2	13±2	13±2	0.834
**Echocardiographic measurements**				
LVEDD, mm (796)	63 (57-70)	62 (55-68)	64 (58-70)	0.045
LVESD, mm (711)	53 (46-60)	52 (44-59)	53 (47-61)	0.043
LVEF, % (859)	29 (24-34)	30 (25-35)	28 (24-33)	0.015
**Medications**				
Beta-blockers (930)	825 (89)	295 (90)	530 (88)	0.518
MRA (927)	602 (65)	202 (61)	400 (67)	0.114
Amiodarone (927)	264 (28)	69 (21)	195 (33)	<0.001
OAC (919)	270 (29)	93 (29)	177 (30)	0.705

The value (in parenthesis) after a feature's name indicates the number of patients with available data. If there is no value reported, data were available for all patients. Continuous variables are expressed as mean ± standard deviation or median (interquartile range), whereas categorical variables are reported as frequencies (n) and percentages (%). The characteristics of the CRT-P and CRT-D groups were compared using unpaired Student's t-test or Mann-Whitney U test for continuous variables and Chi-squared or Fisher's exact test for categorical variables, as appropriate.

Abbreviations as in Supplemental Tables 1 and 2.

Supplemental Table 11 Pairwise log-rank tests for the comparison of the survival of the phenogroups

vs.	Phenogroup 1	Phenogroup 2	Phenogroup 3	Phenogroup 4
**Phenogroup 2**	p=0.063			
**Phenogroup 3**	p=0.120	p=1.000		
**Phenogroup 4**	p<0.001	p<0.001	p<0.001	
**Phenogroup 5**	p<0.001	p<0.001	p<0.001	p=0.140

Endpoint: all-cause death.

Supplemental Table 12 Pairwise log-rank tests for the comparison of the event-free survival of the phenogroups

vs.	Phenogroup 1	Phenogroup 2	Phenogroup 3	Phenogroup 4
**Phenogroup 2**	p=0.071			
**Phenogroup 3**	p=0.160	p=0.870		
**Phenogroup 4**	p<0.001	p<0.001	p<0.001	
**Phenogroup 5**	p<0.001	p<0.001	p<0.001	p=0.150

Endpoint: composite of all-cause death, heart transplantation, and left ventricular assist device implantation.

A **All patients**

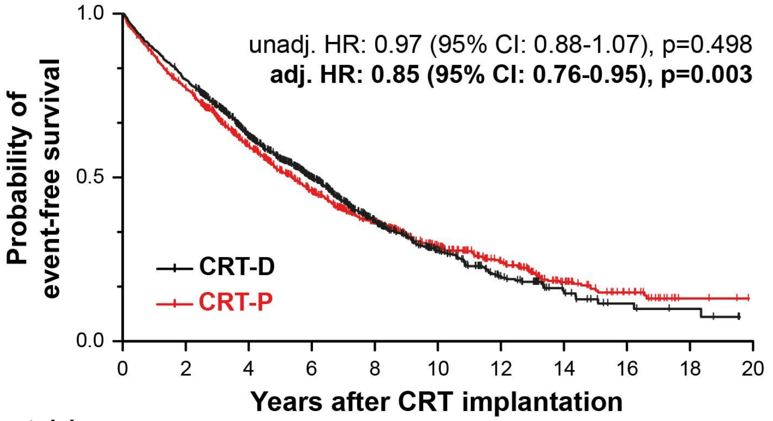

No. at risk

	0	2	4	6	8	10	12	14	16	18	20
CRT-D	1,414	1,126	750	444	213	109	51	19	7	4	-
CRT-P	1,189	908	622	424	278	170	104	44	21	3	-

B **Ischemic patients**

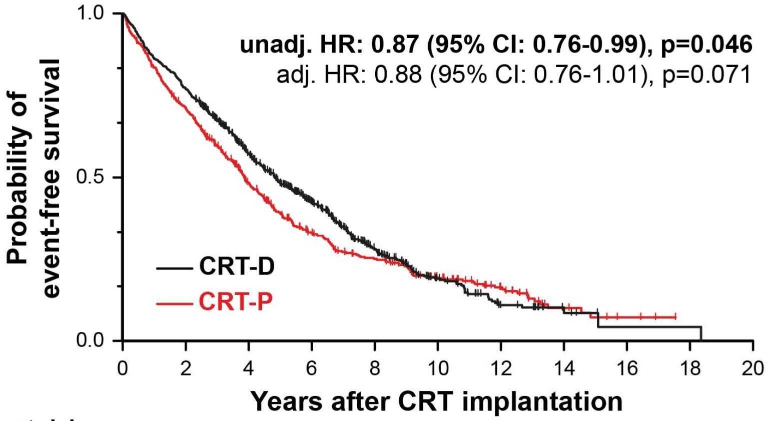

No. at risk

	0	2	4	6	8	10	12	14	16	18	20
CRT-D	817	628	399	234	106	48	17	6	1	1	-
CRT-P	456	321	197	120	84	49	28	8	3	-	-

C **Non-ischemic patients**

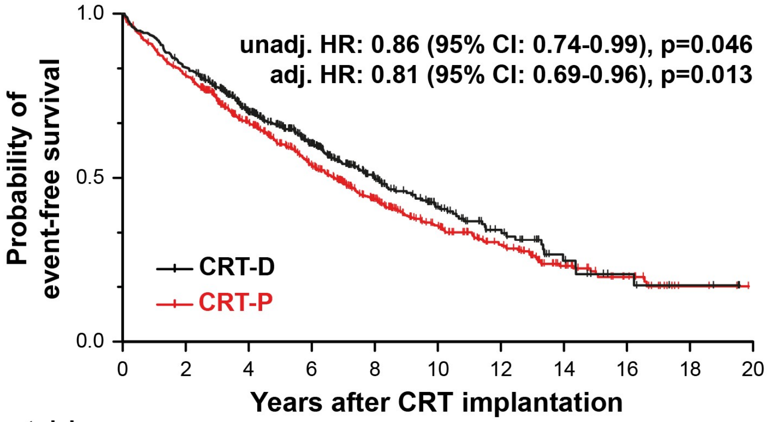

No. at risk

	0	2	4	6	8	10	12	14	16	18	20
CRT-D	597	498	351	210	107	61	34	13	6	3	-
CRT-P	733	587	425	304	194	121	76	36	18	3	-

Supplemental Figure 1 Kaplan-Meier estimates of the time to the composite endpoint of all-cause death, heart transplantation, and left ventricular assist device implantation in the entire study cohort, ischemic patients, and non-ischemic patients

Univariable and multivariable Cox proportional hazards models were used to compute hazard ratios with 95% confidence intervals. Each multivariable model included the following features: device type, age, sex, history of atrial fibrillation, and history of ventricular arrhythmia.

CI – confidence interval, CRT – cardiac resynchronization therapy, CRT-D – cardiac resynchronization therapy defibrillator, CRT-P – cardiac resynchronization therapy pacemaker, HR – hazard ratio

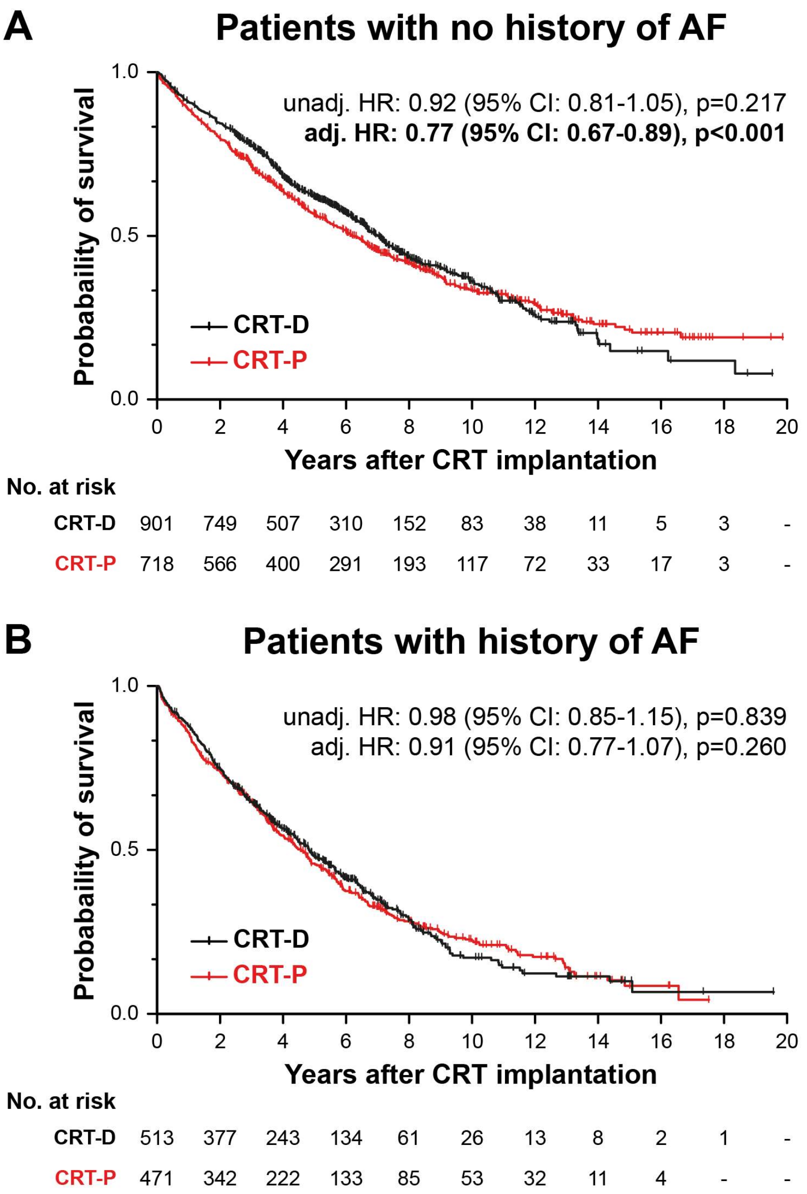

Supplemental Figure 2 Kaplan-Meier estimates of the time to death from any cause in patients with and with no history of atrial fibrillation

Univariable and multivariable Cox proportional hazards models were used to compute hazard ratios with 95% confidence intervals. Each multivariable model included the following features: device type, age, sex, and history of ventricular arrhythmia.

AF – atrial fibrillation, CI – confidence interval, CRT – cardiac resynchronization therapy, CRT-D – cardiac resynchronization therapy defibrillator, CRT-P – cardiac resynchronization therapy pacemaker, HR – hazard ratio

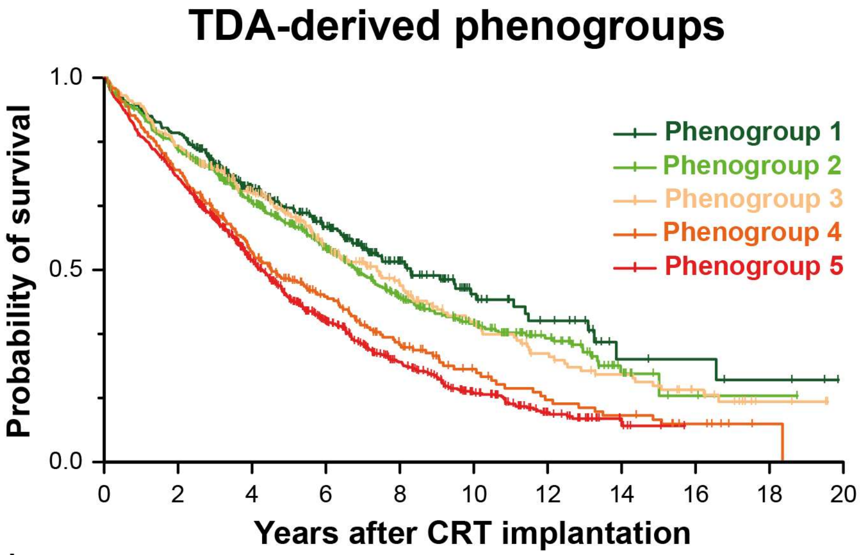

No. at risk											
Phenogroup 1	321	275	186	127	62	30	19	6	5	3	-
Phenogroup 2	553	442	323	213	126	80	43	11	2	1	-
Phenogroup 3	283	232	175	112	72	46	32	23	14	3	-
Phenogroup 4	254	193	129	78	46	25	16	12	6	1	-
Phenogroup 5	964	709	433	251	135	67	28	6	-	-	-

Supplemental Figure 3 Kaplan-Meier estimates of the time to the composite endpoint of all-cause death, heart transplantation, and left ventricular assist device implantation in the five phenogroups identified using topological data analysis

CRT – cardiac resynchronization therapy, TDA – topological data analysis

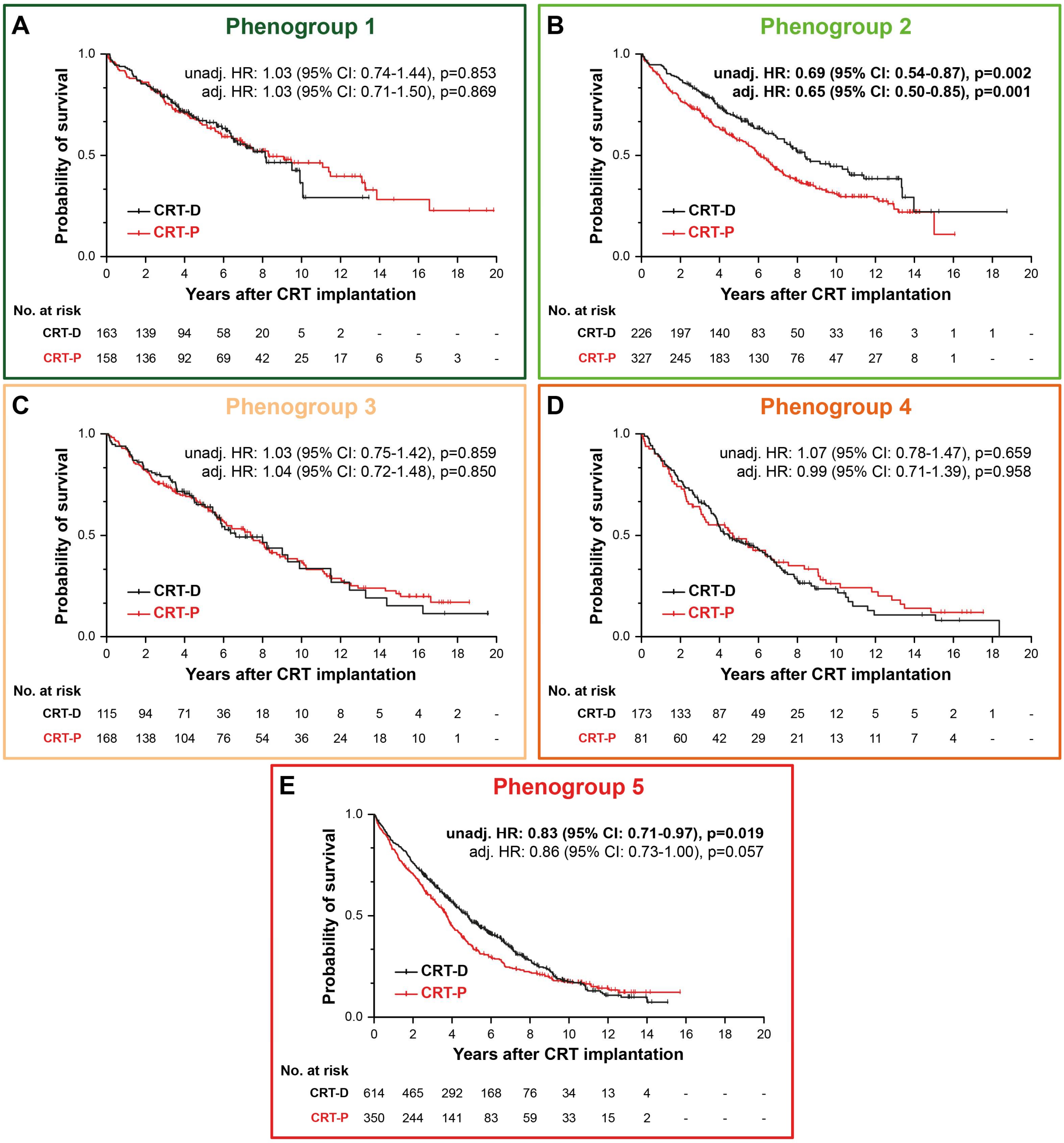

Supplemental Figure 4 Kaplan-Meier estimates of the time to the composite endpoint of all-cause death, heart transplantation, and left ventricular assist device implantation in CRT-D and CRT-P patients in the five phenogroups identified using topological data analysis

Univariable and multivariable Cox proportional hazards models were used to compute hazard ratios with 95% confidence intervals. Each multivariable model included the following features: device type, age, sex, history of atrial fibrillation, and history of ventricular arrhythmia.

CI – confidence interval, CRT – cardiac resynchronization therapy, CRT-D – cardiac resynchronization therapy defibrillator, CRT-P – cardiac resynchronization therapy pacemaker, HR – hazard ratio
